# SO MANY STAKEHOLDERS, SO MANY CONTRACTS!

**DOI:** 10.1093/geroni/igad104.1573

**Published:** 2023-12-21

**Authors:** Marguerite DeLiema

**Affiliations:** University of Minnesota, Twin Cities, Saint Paul, Minnesota, United States

## Abstract

Elder financial fraud is a growing challenge for adult protective services (APS), financial institutions, and state and federal law enforcement agencies that seek effective interventions to protect older adults from continued financial loss. One solution is temporary account holds that prevent suspicious withdrawals from an older or vulnerable adult’s account while investigation and additional interventions are put in place. With funding from the Administration for Community Living, this project seeks to understand the outcomes of temporary account holds on elder safety and financial autonomy in Minnesota. We first worked to improve communication between APS and other Minnesota state agencies to ensure that cases are properly referred and that protection activities are coordinated to achieve the best outcomes for the older adult. Like most collaborative research projects that involve university researchers and state agencies, significant legal agreements were necessary. Multiple subawards and cost share agreements were drafted, data use agreements were created, and IRB approvals from multiple entities were sought. In this presentation, Dr. DeLiema will share lessons learned about how researchers can better prepare for the administrative burden and delayed starts associated with completing these legal administrative processes. She will describe common areas of conflict and competing interests between state agencies and universities around data privacy and ownership, strategies for finding common ground, and possible workarounds to fulfill project aims. Dr. DeLiema will also highlight how to “pitch” a collaboration to community stakeholders using a value proposition that outlines the benefits of the project to the stakeholder.

